# Epidemiology of psychiatric disorders in Texas prisons from 2016 to 2023

**DOI:** 10.1017/S2045796025100267

**Published:** 2025-10-14

**Authors:** Rocksheng Zhong, Myrna Serna, Jeffrey Farroni, Biai Digbeu, Gwen Baillargeon, John Pulvino, Joseph Penn, Owen Murray, Jacques Baillargeon

**Affiliations:** 1 Department of Psychiatry and Behavioral Sciences, University of Texas Medical Branch, Galveston, TX, USA; 2 Department of Internal Medicine, University of Texas Medical Branch, Galveston, TX, USA; 3 Institute for Bioethics and Health Humanities, School of Public and Population Health, University of Texas Medical Branch, Galveston, TX, USA; 4 Department of Biostatistics and Data Science, University of Texas Medical Branch, Galveston, TX, USA; 5 Correctional Managed Care, University of Texas Medical Branch, Galveston, TX, USA; 6 Department of Public Health, Robbins College of Health and Human Sciences, Baylor University, Waco, TX, USA

**Keywords:** Correctional psychiatry, epidemiology, forensic psychiatry, mental disorders, mood disorders, prisons, psychotic disorders

## Abstract

**Aims:**

Although the United States incarcerates nearly two million people, the epidemiology of psychiatric disorders in correctional populations is not well understood, and no study has examined temporal trends in psychiatric disorder prevalences within a single correctional system. This study assessed how psychiatric disorder prevalences have changed in the Texas Department of Criminal Justice (TDCJ), the largest American state prison system housing post-conviction, sentenced individuals.

**Methods:**

This retrospective cohort study of TDCJ electronic medical record data from 1 January 2016 through 31 December 2023 included all persons incarcerated for any duration during that period. Diagnoses were based on International Classification of Disease (ICD-10) diagnostic codes. Outcomes were annual prevalences of depressive, bipolar and schizophrenia spectrum disorders stratified by age, race and sex. Cochran-Armitage Tests were used to assess temporal trends within each stratum. Two-way interactions were assessed by fitting Generalized Estimating Equations models using autoregressive correlation with repeated subjects.

**Results:**

The overall population ranged from 170,269 to 222,798 individuals. Approximately, one-third were White (34.5–35.4%), one-third Black (31.0–32.3%), and one-third Hispanic (32.7–33.5%). Most were aged 30–49 (52.8–57.3%), and men (88.9–90.7%) outnumbered women (9.3–11.1%). The prevalences (per 100 [95% CI]) of psychiatric disorders generally increased when comparing 2016 to 2023. Depressive disorders increased the most among those aged 30–49 (5.23 [5.10–5.35] to 6.71 [6.56–6.86]), Hispanic individuals (3.86 [3.72–4.00] to 5.72 [5.53–5.90]), and men (4.72 [4.63–4.82] to 6.53 [6.42–6.65]). Bipolar disorders increased the most among those aged ≥50 (2.57 [2.42–2.72] to 3.46 [3.29–3.63]), Hispanic individuals (1.31 [1.23–1.40] to 2.23 [2.11–2.35]), and men (2.26 [2.20–2.33] to 3.12 [3.04–3.20]). Schizophrenia spectrum disorders increased the most among those aged ≤29 (1.33 [1.24–1.42] to 2.52 [2.35–2.68]), Hispanic individuals (1.53 [1.44–1.62] to 3.21 [3.35–4.40]), and women (1.27 [1.56–1.89] to 4.24 [3.95–4.53]). When stratified by demographic variables, trend tests were significant for nearly all comparisons (*P* < 0.0001), and all two-way interactions were significant (*P* < 0.0001).

**Conclusions:**

The prevalences of major psychiatric disorders in the Texas prison system increased when comparing 2016 to 2023, with certain disorders rising more rapidly than others within specific subgroups. These findings emphasize the need for expanded mental health treatment options and resources within correctional settings.

## Introduction

The United States incarcerates nearly two million people, or approximately one out of every 200 Americans annually (Dlugacz, 2014; Sawyer and Wagner, 2023). Americans make up one-fifth of all incarcerated people in the world, equal to the number of people incarcerated by 194 other countries combined (Sawyer and Wagner, 2023). Numerous scholars have described mass incarceration as a public health crisis, given not only its negative effects on a variety of health outcomes, including infectious diseases, chronic illness, life expectancy, mental health, and addiction, but also its role in exacerbating health inequities (Cloud *et al.*, 2023; Dumont *et al.*, 2012; Wang and Shavit, 2023; Wildeman and Wang, 2017).

Following the deinstitutionalization movement in the United States during the 1960s and 1970s, in which long-term psychiatric hospitals were closed in favour of community care that never fully materialized, correctional institutions have become the de facto primary mental health providers for many people with serious mental illness (typically depressive, bipolar, and schizophrenia spectrum disorders) (Sisti *et al.*, 2015). Although estimates vary widely depending on sampling and assessment methods, it is well-known that people with mental illness are overrepresented in correctional settings (Diamond *et al.*, 2001). In one study, twice as many people with mental illness were reported in correctional settings compared to their community counterparts (Diamond *et al.*, 2001). Other studies of correctional institutions have estimated the rates of serious mental illness at about 8–19%, or alternatively, 15–20% requiring psychiatric treatment during incarceration (Metzner, 2002). A 2024 umbrella review of 17 meta-analyses found that 3.7% of incarcerated individuals had psychotic illnesses, and 11.4% had major depression (Favril *et al.*, 2024).

However, scant research has examined how rates of major psychiatric disorders have changed over time within a single correctional system, and such studies have not extended beyond 2019 (Al-Rousan *et al.*, 2017; Baillargeon *et al.*, 2009b; Browne *et al.*, 2023; Bukten *et al.*, 2024; Butler *et al.*, 2022; Fazel and Danesh, 2002; Prins, 2014). Examining recent temporal trends is particularly valuable in the context of the changing landscape of criminal justice policies, the onset of the COVID-19 pandemic in 2020, and shifts in the epidemiology of mental illness in the general population. A greater understanding of these changes is critical for effective allocation of resources to address important mental health needs. Given the size of the correctional population in the United States, even small, incremental improvements in care can significantly affect the health and public health of incarcerated persons (Massoglia and Remster, 2019). Accordingly, we investigated the rates of serious mental illness from 2016 through 2023 in the Texas Department of Criminal Justice (TDCJ), the nation’s largest state prison system, which houses post-conviction, sentenced individuals.

## Methods

### Design and study sample

This retrospective cohort study included all individuals incarcerated in TDCJ from 1 January 2016, through 31 December 2023. TDCJ consists of 103 prison units distributed across Texas. Eight units are privately operated, and the remainder are state-operated. Twelve units house women only, three are co-gender, and 88 are men-only. Specialized programmes and units exist for people with developmental disabilities, substance use disorders, severe psychiatric disorders and acute medical problems, as well as one dedicated geriatric facility (Texas Department of Criminal Justice, 2025). All individuals incarcerated in TDCJ are post-conviction and serving criminal sentences. In Fiscal Year 2023 (1 September 2022 through 31 August 2023), TDCJ received 53,852 individuals: 17,109 were convicted of violent offenses, 9,184 of property offenses, 15,178 of drug offenses and 12,382 of other offenses. The average length of sentence was 6.8 years (Texas Department of Criminal Justice, 2023b). During this period, TDCJ had a total operating budget of over $3.5 billion (Texas Department of Criminal Justice, 2023a).

All persons incarcerated in TDCJ are required to undergo medical and mental health examinations at the time of intake. Those declining to participate in the intake examination are not permitted to enter their cells until the examinations are completed. Individuals unable to participate due to illness are referred to an acute care facility for stabilization before returning to TDCJ to complete the intake process. Consequently, 100% of individuals incarcerated in TDCJ complete the intake examination. The evaluation lasts approximately 60 minutes and consists of a detailed history, comprehensive physical exam and any appropriate laboratory testing. All diagnoses are made by physicians or advanced practice providers during the initial evaluation and/or subsequent medical encounters and are classified according to the International Classification of Diseases, Tenth Revision (ICD-10) coding system. These data, along with demographic information, are maintained in an institution-wide electronic medical record (EMR) that is routinely updated to reflect patients’ current health status. For this study, EMR data were extracted and organized into eight sequential calendar-year cohorts for time trend analysis. An individual was included in a calendar-year cohort if they were incarcerated for at least one day during that year. The study was reviewed and approved by the University of Texas Medical Branch (UTMB) Institutional Review Board and the TDCJ Research and Development Department.

The study was designed to compare the prevalence over time of major psychiatric disorders that make up the usual set of serious mental illnesses: depressive disorders, bipolar disorders and schizophrenia spectrum disorders. Depressive disorders (F32–F33) included major depressive disorder, depressive disorder due to a medical condition and unspecified depressive disorder. Bipolar disorders (F30–F31) included bipolar I disorder, bipolar II disorder and unspecified bipolar disorder. Schizophrenia spectrum disorders (F20–F29, F60.1) included schizophrenia, schizoaffective disorder, delusional disorder, schizophreniform disorder, brief psychotic disorder and unspecified psychotic disorder. Although ICD-10 codes permit more specific diagnostic differentiation, we grouped psychiatric disorders into major diagnostic categories to enhance diagnostic validity and reproducibility. A person was considered to have a disorder if their diagnosis date occurred before or during the calendar-year cohort in which they were included. Demographic information consisted of race (White, Black, Hispanic and Other) and sex (male and female), which were based on self-report at intake, as well as age (≤29 years, 30–49 years and ≥50 years), which was calculated from date of birth. Thus, both diagnostic and demographic variables were current up to a given calendar year but did not include any updates that post-dated that year.

### Statistical analyses

All statistical analyses were performed using SAS software, Version 9.4 (SAS Institute Inc., Cary, NC). Overall yearly summary demographics stratified by age, race and sex were calculated. Yearly prevalences for each psychiatric disorder diagnosis, as well as yearly prevalences stratified by age, race and sex within each diagnosis, were calculated. A person was included in the calendar-year cohort if they were incarcerated in TDCJ for at least one day during that year. Trend tests using the Cochran-Armitage Test for Trend were performed to assess whether prevalences increased over time within each respective diagnosis stratum. For each diagnosis, two-way interactions between year/age, year/race and year/sex were assessed by fitting Generalized Estimating Equations models using autoregressive (AR1) correlation with repeated subjects. In each model, the outcome was the diagnosis of interest (yes/no), and each interaction term was assessed one at the time.

## Results

Demographic characteristics of the study population are presented in Table 1. The number of persons incarcerated in TDCJ decreased steadily from 222,798 in 2016 to 170,269 in 2021. The decline was accelerated by a three-month halt on new admissions in 2020 in the wake of COVID-19 before intakes were resumed at a reduced capacity. It was not until 2022 that the TDCJ population began to increase again, reaching 185,451 by 2023. Throughout the study period, racial and sex distributions remained largely constant. The population divided roughly into one-third White, one-third Black and one-third Hispanic, with other racial groups making up less than 1% of the sample. Men outnumbered women by a 9:1 ratio. Most individuals were aged 30–49 years. This group, along with those aged 50 years or older, grew during the study period, while the proportion of persons aged 29 years or younger diminished.

**Table 1.** Demographic characteristics

		Age			Race				Sex	
Year	Total	≤29	30−49	≥50	White	Black	Hispanic	Other	Male	Female
2016	222,798	61,090 (27.4%)	117,634 (52.8%)	44,074 (19.8%)	76,835 (34.5%)	71,805 (32.2%)	72,966 (32.7%)	1,192 (0.5%)	198,134 (88.9%)	24,664 (11.1%)
2017	220,669	58,565 (26.5%)	117,629 (53.3%)	44,475 (20.2%)	76,585 (34.7%)	70,138 (31.8%)	72,759 (33.0%)	1,187 (0.5%)	196,465 (89.0%)	24,204 (11.0%)
2018	219,742	55,825 (25.4%)	118,584 (54.0%)	45,333 (20.6%)	77,251 (35.2%)	68,841 (31.3%)	72,460 (33.0%)	1,190 (0.5%)	195,678 (89.0%)	24,064 (11.0%)
2019	214,440	51,714 (24.1%)	117,252 (54.7%)	45474 (21.2%)	75,856 (35.4%)	66,498 (31.0%)	70,926 (33.1%)	1,160 (0.5%)	190,919 (89.0%)	23,521 (11.0%)
2020	182,448	40,138 (22.0%)	100,605 (55.1%)	41,705 (22.9%)	64,230 (35.2%)	57,057 (31.3%)	60,154 (33.0%)	1,007 (0.6%)	164,427 (90.1%)	18,021 (9.9%)
2021	170,269	35,399 (20.8%)	95,049 (55.8%)	39,821 (23.4%)	59,798 (35.1%)	53,159 (31.2%)	56,333 (33.1%)	979 (0.6%)	154,373 (90.7%)	15,896 (9.3%)
2022	176,798	35,418 (20.0%)	100,308 (56.7%)	41,072 (23.2%)	62,204 (35.2%)	54,840 (31.0%)	58,743 (33.2%)	1,011 (0.6%)	159,625 (90.3%)	17173 (9.7%)
2023	185,451	35,894 (19.4%)	106,173 (57.3%)	43,384 (23.4%)	64,546 (34.8%)	57,763 (31.1%)	62,074 (33.5%)	1,068 (0.6%)	167,007 (90.1%)	18,444 (9.9%)

**Fig. 1.**
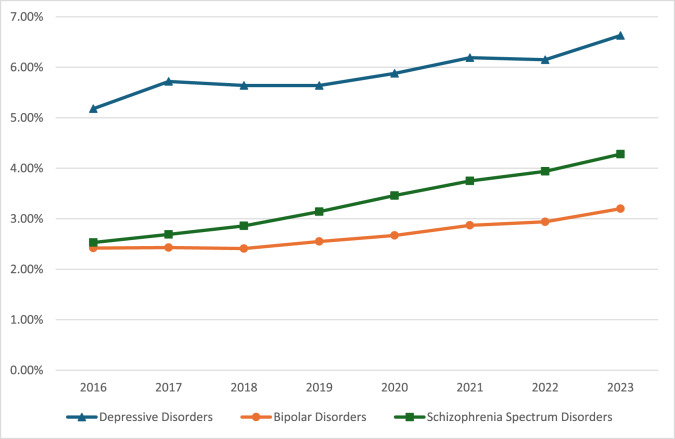
Prevalence of psychiatric disorders.

Depressive disorders (5.18–6.63%) were more prevalent than bipolar disorders (2.42–3.20%) and schizophrenia spectrum disorders (2.53–4.28%) (Table 2 and Fig. 1). All disorders demonstrated a general trend towards rising prevalence, with schizophrenia spectrum disorders increasing the most (69% relative rate increase), followed by bipolar disorders (32%) and depressive disorders (28%).

**Table 2.** Prevalence of psychiatric disorders

Year	Depressive disorders	Bipolar disorders	Schizophrenia spectrum disorders
2016	11,546 (5.18%)	5,400 (2.42%)	5,640 (2.53%)
2017	12,616 (5.72%)	5,355 (2.43%)	5,943 (2.69%)
2018	12,388 (5.64%)	5,306 (2.41%)	6,279 (2.86%)
2019	12,093 (5.64%)	5,464 (2.55%)	6,734 (3.14%)
2020	10,722 (5.88%)	4,868 (2.67%)	6,310 (3.46%)
2021	10,547 (6.19%)	4,887 (2.87%)	6,392 (3.75%)
2022	10,871 (6.15%)	5,202 (2.94%)	6,974 (3.94%)
2023	12,299 (6.63%)	5,936 (3.20%)	7,940 (4.28%)

**Fig. 2:**
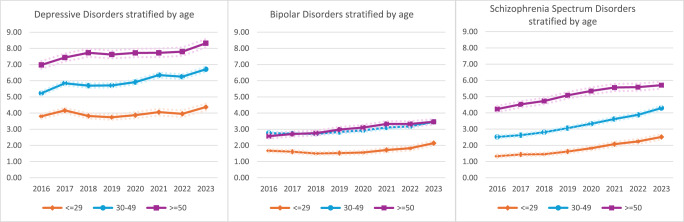
Prevalence of psychiatric disorders stratified by age.^a^

^a^The dotted lines denote the 95% CI.

**Fig. 3.**
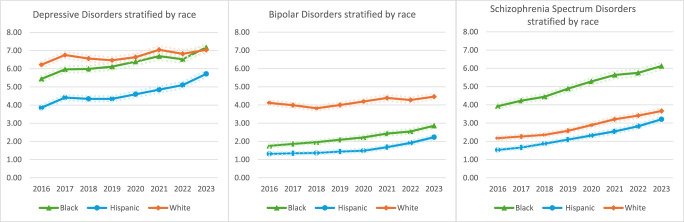
Prevalence of psychiatric disorders stratified by race.^a^

^a^The dotted lines denote the 95% CI.

**Fig. 4.**
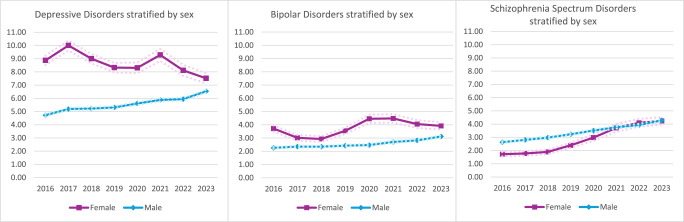
Prevalence of psychiatric disorders stratified by sex.^a^

^a^The dotted lines denote the 95% CI.

Figures 2–4 show the data stratified by age, race and sex. Cochran-Armitage Tests for Trend were significant for all comparisons at a level of *P* < 0.0001, except for four strata: The Other racial category showed statistically significant time trends for depressive (*P* = 0.0036) and schizophrenia spectrum (*P* = 0.0175) disorders but not bipolar disorders (*P* = 0.1213), most likely due to the low numbers of people in this category. The ≤29 years age category showed a statistically significant time trend for depressive disorders (*P* = 0.0055).

Psychiatric disorders were about 1.5–2 times more common among the oldest persons in the sample compared to those aged 29 years or less. White (6.22–7.04%) and Black (5.44–7.17%) individuals had consistently higher rates of depressive disorders than Hispanic (3.86–5.72%) individuals over time. White (4.12–4.46%) individuals also had higher rates of bipolar disorders than both Black (1.75–2.86%) and Hispanic (1.31–2.23%) individuals. Meanwhile, Black (3.93–6.13%) individuals had higher rates of schizophrenia spectrum disorders than White (2.17–3.66%) and Hispanic (1.53–3.21%) individuals. When the data were stratified by sex, women had a higher prevalence of mood disorders but unlike men, did not demonstrate a steady upward trend over time.

In addition, all two-way interactions between year/age, year/race and year/sex were significant (*P* < 0.0001; see also Supplemental Tables 3–5 in the Appendix). In other words, the time trends were modified by age, race, and sex. The prevalence of depressive disorders increased by 15% and 19% among the youngest and oldest incarcerated persons, respectively, compared to 28% among those who were middle-aged; increased by 32% and 48% among Black and Hispanic individuals, respectively, compared to only 13% among White individuals; and increased by 38% among men compared to a 15% decrease among women. The prevalence of bipolar disorders increased by 27% among the youngest group, 25% among the middle-aged group and 35% among the oldest group; increased by 63% among Black individuals and 70% among Hispanic individuals but only 8% among White individuals; and increased by 37% among men but only 5% among women. Lastly, the prevalence of schizophrenia spectrum disorders increased by 89% in the youngest group and 71% in the middle-aged group compared to 35% in the oldest group; increased by 56% among Black individuals, 68% among White individuals and 110% among Hispanic individuals; and increased by 146% among women compared to 63% among men.

## Discussion

We found that when comparing 2016 to 2023, the prevalence of major psychiatric disorders in TDCJ increased in a linear fashion by about 30% for mood disorders and about 70% for schizophrenia spectrum disorders, reaching peaks of 6.63% for depressive disorders, 3.20% for bipolar disorders and 4.28% for schizophrenia spectrum disorders. This trend persisted independent of changing admissions policies in the wake of COVID-19. Older incarcerated individuals had higher rates of all three classes of psychiatric disorders, and women had higher rates of mood disorders.

Similar to previous analyses of racial disparities among non-incarcerated individuals, our results indicate that White individuals incarcerated in TDCJ had higher rates of bipolar disorders, and Black individuals incarcerated in TDCJ had higher rates of schizophrenia spectrum disorders (Haeri *et al.*, 2011; Schwartz and Blankenship, 2014). Research has consistently shown that across inpatient and outpatient settings, Black people are more likely to be diagnosed with schizophrenia spectrum disorders, while White people are more likely to be diagnosed with bipolar disorders (Schwartz and Blankenship, 2014). These patterns are thought reflect differences in how symptoms are reported by Black versus White individuals, how clinicians may interpret those symptoms, and discrepancies in help-seeking behaviours and access to care (Haeri *et al.*, 2011). Studies of racial disparities in depressive disorders, however, have been more mixed. A 2007 review identified two studies reporting higher rates of depression among White compared to Black or Hispanic individuals, one study showing higher rates among Hispanic compared to White and Black individuals, and one study reporting no significant differences between the three groups (Simpson *et al.*, 2007). In our sample, Hispanic individuals incarcerated in TDCJ had lower rates than White and Black individuals.

Finally, women exhibited more inconsistent patterns of mental illness over time, though this finding may be partly attributable to their relatively small numbers, as they made up only about one-tenth of the incarcerated population. At the same time, our sample sizes ranging from 15,896 to 24,664 women were robust in absolute terms, and the relevant interaction effects were statistically significant. It is unclear why the prevalence of depression in particular decreased among women during the study period. Notably, when comparing 2023 to 2016, the total number of incarcerated women declined more sharply than men (25.2% vs 15.7% decrease), and as a result, the proportion of men to women increased slightly, from about 89% to over 90%. One theory, which remains highly speculative, may be that as the number of women declined, women with depression were seen as particularly low risk and therefore more likely to be released than women with bipolar or schizophrenia spectrum disorders and more likely to be released than men.

Overall, our findings are consistent with established research concerning the prevalence of psychiatric disorders in correctional settings. Several narrative and systematic reviews have assessed the prevalence of depressive disorders to range from 3% to 29%, bipolar disorders from 1% to 16%, and schizophrenia spectrum disorders from 2% to 7% (Diamond *et al.*, 2001 ; Fazel and Baillargeon, 2011; Fazel and Danesh, 2002; Prins, 2014). Meta-analyses have estimated the prevalence of depressive disorders at 11–12% and schizophrenia spectrum disorders at about 4% (Favril *et al.*, 2024; Fazel and Seewald, 2012). A prior study of the TDCJ population from September 2006 to September 2007 observed comparable prevalences of major depressive disorder (4.2%), bipolar disorder (2.6%) and schizophrenia spectrum disorders (3.8%) (Baillargeon *et al.*, 2009b). Our results fell within the lower to middle ranges of these estimates and were likely conservative, given that we relied on specialized clinician assessments recorded in the EMR rather than self-report or screening tools. Furthermore, we combined diagnoses within the same broad categories to minimize diagnostic bloat and excessive comorbidity. This approach also accounted for potential diagnostic revisions and updates as clinicians obtained more information over time, as well as any potential artefactual effects owing to changes in diagnostic coding schemes.

Our findings are also consistent with observed increases in the prevalence of serious mental illnesses in the general population. Over the last three decades, rates of depression and bipolar disorder have risen (Ferrari *et al.*, 2016; Weinberger *et al.*, 2018; Zutshi *et al.*, 2011), particularly among adolescents and young adults (Mojtabai *et al.*, 2016). For instance, using data from the National Survey on Drug Use and Health, which surveys non-institutionalized Americans aged 12 and older, Weinberger *et al.* (2018), found that from 2005 to 2015, the prevalence of past-year depression rose from 6.62% to 7.28%. In an Australian sample, 2.9% of respondents to the South Australian Health Omnibus Surveys reported doctor-diagnosed bipolar disorder in 2008, compared to 1.7% in 2004 and 1.1% in 1998 (Zutshi *et al.*, 2011). Lai *et al.* (2024) estimated that the number of prevalent cases of bipolar disorder increased globally by 59.3% between 1990 and 2019, though rates varied according to country and age. Data on the prevalence of schizophrenia spectrum disorders are more limited and inconsistent; however, a systematic review found a median 12-month prevalence of 0.33% for studies before 1999, compared to 0.46% for studies from 2000 to 2009 (Simeone *et al.*, 2015). COVID-19 has further exacerbated these trends (Winkler *et al.*, 2020; Wu *et al.*, 2021).

The present research highlights the critical importance of psychiatric treatment in correctional settings. Our study is the first of its kind to demonstrate the persistent and growing need for mental health services and resources over time within a large state-wide prison system, especially amid the backdrop of the COVID-19 pandemic. We confirmed that not only are people with serious mental illness already overrepresented in prisons, but their numbers have also increased.

These shifts may be driven by both higher numbers of admissions and fewer releases of people with serious mental illness. Public mental health systems remain underfunded and under-resourced, and people with untreated serious mental illness are at higher risk for criminal justice involvement (Gottfried and Christopher, 2017; Hirschtritt and Binder, 2017). Once incarcerated, they may have difficulty complying with prison regulations, resulting in additional violations or offenses that can lengthen their sentences. After release from prison, people with serious mental illness may be more likely to recidivate or violate the terms of their early release (Baillargeon *et al.*, 2009a). In states that implement progressively harsher punishments for people who repeatedly offend (i.e., three-strikes laws), mental illness associated with multiple convictions may lead to enhanced classification and longer sentences for what would otherwise be considered minor crimes.

Our results must be interpreted in light of several potential limitations. First, comparisons between prison and community samples should be made cautiously since prison populations are composed disproportionately of men, younger individuals, and people from racial and ethnic minorities. Moreover, although our findings were consistent with previous research examining American racial and ethnic disparities, the study may be less generalizable to other countries with racial and ethnic compositions that differ dramatically from the United States. Second, although TDCJ employs universal, standardized mental health screening and evaluation procedures, less severe psychiatric disorders such as anxiety and personality disorders are not as rigorously evaluated in TDCJ; therefore, we restricted our analyses to major categories of serious mental illness. Even so, some individuals may have been misdiagnosed, or some data may have been entered incorrectly into the EMR. In short, similar to other studies using TDCJ EMR data, our findings depend on the reliability and validity of the evaluations and diagnoses of mental health professionals in TDCJ (Baillargeon *et al.*, 2000, 2001, 2009a, 2009b; Baillargeon and Contreras, 2001). At the same time, this may also be a strength of the study, since EMR data reflect real-world diagnosis and treatment of people with serious mental illness. Third, the TDCJ population is highly dynamic, with substantial turnover as people are admitted, paroled, discharged outright, or reincarcerated for violations or new offenses. Ideally, we would have traced individuals through the system over time to capture the evolution of their mental health diagnoses, but our dataset did not permit this level of tracking. Instead, individuals were included in a calendar-year cohort if they were incarcerated in TDCJ for any period of time during that year. Nevertheless, our diagnostic categories should be relatively stable and accurate since we considered broad classes of serious mental illnesses, which are usually chronic over multiple years.

In conclusion, we characterized the epidemiology of major psychiatric disorders in TDCJ, the largest state prison system in the United States. We showed that the prevalence of major psychiatric disorders in prison has continued to rise since 2016. Addressing this ongoing challenge will require renewed efforts to divert individuals with serious mental illness from the criminal justice system on the front end, enhance treatment within correctional settings, and improve coordination with community mental health systems for post-release care and support.

## Supporting information

10.1017/S2045796025100267.sm001 Zhong et al. supplementary material Zhong et al. supplementary material

## Data Availability

Data will not be shared due to the vulnerable population involved in this research and the TDCJ requirement that a research agreement must exist between TDCJ and any non-TDCJ entity before any data are shared.

## Supplementary material

The supplementary material for this article can be found at https://doi.org/10.1017/S2045796025100267.
