# Supplementary material for: Epidemiology of psychiatric disorders in Texas prisons from 2016 to 2023
Source: Epidemiol Psychiatr Sci. 2025 Oct 14;34:e51. doi: 10.1017/S2045796025100267 (PMC12555086; doi:10.1017/S2045796025100267)
Supplement: Zhong et al. supplementary material [file S2045796025100267sup001.docx]

Supplementary Materials

| **Supplemental Table 1.1: Psychotic Disorders prevalence rates stratified by race** | | | |  |  |  |  |  |  |
| --- | --- | --- | --- | --- | --- | --- | --- | --- | --- |
|  | **Year** | | | | | | | |  |
| **Race Prevalence (%)** | 2016 | 2017 | 2018 | 2019 | 2020 | 2021 | 2022 | 2023 | **P-value for Cochran-Armitage Trend test** |
| Black | 3.93 | 4.23 | 4.44 | 4.89 | 5.28 | 5.64 | 5.75 | 6.13 | <.0001 |
| Hispanic | 1.53 | 1.66 | 1.87 | 2.09 | 2.32 | 2.54 | 2.82 | 3.21 | <.0001 |
| Other | 2.85 | 3.20 | 3.78 | 3.88 | 4.47 | 4.39 | 4.06 | 4.40 | 0.0175 |
| White | 2.17 | 2.26 | 2.35 | 2.58 | 2.89 | 3.21 | 3.41 | 3.66 | <.0001 |
| *Interaction term between year and race was significant, P < 0.0001. |  |  |  |  |  |  |  |  |  |
|  |  |  |  |  |  |  |  |  |  |
| **Supplemental Table 1.2: Depressive Disorders prevalence stratified by race** | | | |  |  |  |  |  |  |
|  | **Year** | | | | | | | |  |
| **Race Prevalence (%)** | 2016 | 2017 | 2018 | 2019 | 2020 | 2021 | 2022 | 2023 | **P-value for Cochran-Armitage Trend test** |
| Black | 5.44 | 5.96 | 5.99 | 6.11 | 6.39 | 6.69 | 6.52 | 7.17 | <.0001 |
| Hispanic | 3.86 | 4.41 | 4.34 | 4.34 | 4.59 | 4.85 | 5.10 | 5.72 | <.0001 |
| Other | 3.86 | 4.30 | 4.29 | 3.88 | 4.77 | 4.90 | 5.44 | 6.09 | 0.0036 |
| White | 6.22 | 6.75 | 6.56 | 6.46 | 6.64 | 7.04 | 6.82 | 7.04 | <.0001 |
| *Interaction term between year and race was significant, P < 0.0001. |  |  |  |  |  |  |  |  |  |
|  |  |  |  |  |  |  |  |  |  |
| **Supplemental Table 1.3: Bipolar Disorders prevalence stratified by race** | | | |  |  |  |  |  |  |
|  | **Year** | | | | | | | |  |
| **Race Prevalence (%)** | 2016 | 2017 | 2018 | 2019 | 2020 | 2021 | 2022 | 2023 | **P-value for Cochran-Armitage Trend test** |
| Black | 1.75 | 1.86 | 1.96 | 2.09 | 2.22 | 2.43 | 2.54 | 2.86 | <.0001 |
| Hispanic | 1.31 | 1.34 | 1.36 | 1.44 | 1.48 | 1.68 | 1.92 | 2.23 | <.0001 |
| Other | 1.59 | 1.94 | 1.85 | 1.98 | 1.69 | 2.55 | 2.37 | 2.25 | 0.1213 |
| White | 4.12 | 3.99 | 3.82 | 4.00 | 4.19 | 4.39 | 4.27 | 4.46 | <.0001 |
| *Interaction term between year and race was significant, P < 0.0001. |  |  |  |  |  |  |  |  |  |

| **Supplemental Table 2.1: Psychotic Disorders prevalence stratified by age** | | | |  |  |  |  |  |  |
| --- | --- | --- | --- | --- | --- | --- | --- | --- | --- |
|  | **Year** | | | | | | | |  |
| **Age Group Prevalence (%)** | 2016 | 2017 | 2018 | 2019 | 2020 | 2021 | 2022 | 2023 | **P-value for Cochran-Armitage Trend test** |
| <=29 years | 1.33 | 1.44 | 1.45 | 1.62 | 1.82 | 2.07 | 2.24 | 2.52 | <.0001 |
| 30-49 years | 2.52 | 2.63 | 2.80 | 3.06 | 3.33 | 3.62 | 3.88 | 4.30 | <.0001 |
| >=50 years | 4.23 | 4.53 | 4.73 | 5.08 | 5.35 | 5.56 | 5.58 | 5.70 | <.0001 |
| *Interaction term between year and age was significant, P < 0.0001. |  |  |  |  |  |  |  |  |  |
|  |  |  |  |  |  |  |  |  |  |
|  |  |  |  |  |  |  |  |  |  |
| **Supplemental Table 2.2: Depressive Disorders prevalence stratified by age** | | | |  |  |  |  |  |  |
|  | **Year** | | | | | | | |  |
| **Age Group Prevalence (%)** | 2016 | 2017 | 2018 | 2019 | 2020 | 2021 | 2022 | 2023 | **P-value for Cochran-Armitage Trend test** |
| <=29 years | 3.80 | 4.16 | 3.82 | 3.74 | 3.87 | 4.06 | 3.95 | 4.37 | 0.0055 |
| 30-49 years | 5.23 | 5.84 | 5.69 | 5.71 | 5.91 | 6.35 | 6.25 | 6.71 | <.0001 |
| >=50 years | 6.98 | 7.44 | 7.73 | 7.62 | 7.72 | 7.72 | 7.80 | 8.32 | <.0001 |
| *Interaction term between year and age was significant, P < 0.0001. |  |  |  |  |  |  |  |  |  |
|  |  |  |  |  |  |  |  |  |  |
|  |  |  |  |  |  |  |  |  |  |
| **Supplemental Table 2.3: Bipolar Disorders prevalence stratified by age** | | | |  |  |  |  |  |  |
|  | **Year** | | | | | | | |  |
| **Age Group Prevalence (%)** | 2016 | 2017 | 2018 | 2019 | 2020 | 2021 | 2022 | 2023 | **P-value for Cochran-Armitage Trend test** |
| <=29 years | 1.67 | 1.61 | 1.50 | 1.53 | 1.56 | 1.71 | 1.82 | 2.14 | <.0001 |
| 30-49 years | 2.76 | 2.73 | 2.71 | 2.83 | 2.93 | 3.11 | 3.18 | 3.45 | <.0001 |
| >=50 years | 2.57 | 2.70 | 2.76 | 2.98 | 3.10 | 3.33 | 3.33 | 3.46 | <.0001 |
| *Interaction term between year and age was significant, P < 0.0001. |  |  |  |  |  |  |  |  |  |

| **Supplemental Table 3.1: Psychotic Disorders prevalence stratified by sex** | | | | |  |  |  |  |  |
| --- | --- | --- | --- | --- | --- | --- | --- | --- | --- |
|  | **Year** | | | | | | | |  |
| **Sex Prevalence (%)** | 2016 | 2017 | 2018 | 2019 | 2020 | 2021 | 2022 | 2023 | **P-value for Cochran-Armitage Trend test** |
| Female | 1.72 | 1.78 | 1.90 | 2.39 | 2.98 | 3.71 | 4.09 | 4.24 | <.0001 |
| Male | 2.63 | 2.81 | 2.98 | 3.23 | 3.51 | 3.76 | 3.93 | 4.29 | <.0001 |
| *Interaction term between year and sex was significant, P < 0.0001. |  |  |  |  |  |  |  |  |  |
|  |  |  |  |  |  |  |  |  |  |
|  |  |  |  |  |  |  |  |  |  |
|  |  |  |  |  |  |  |  |  |  |
| **Supplemental Table 3.2: Depressive Disorders prevalence stratified by sex** | | | | |  |  |  |  |  |
|  | **Year** | | | | | | | |  |
| **Sex Prevalence (%)** | 2016 | 2017 | 2018 | 2019 | 2020 | 2021 | 2022 | 2023 | **P-value for Cochran-Armitage Trend test** |
| Female | 8.88 | 10.00 | 9.00 | 8.32 | 8.31 | 9.29 | 8.12 | 7.51 | <.0001 |
| Male | 4.72 | 5.19 | 5.22 | 5.31 | 5.61 | 5.88 | 5.94 | 6.53 | <.0001 |
| *Interaction term between year and sex was significant, P < 0.0001. |  |  |  |  |  |  |  |  |  |
|  |  |  |  |  |  |  |  |  |  |
|  |  |  |  |  |  |  |  |  |  |
|  |  |  |  |  |  |  |  |  |  |
| **Supplemental Table 3.3: Bipolar Disorders prevalence stratified by sex** | | | |  |  |  |  |  |  |
|  | **Year** | | | | | | | |  |
| **Sex Prevalence (%)** | 2016 | 2017 | 2018 | 2019 | 2020 | 2021 | 2022 | 2023 | **P-value for Cochran-Armitage Trend test** |
| Female | 3.72 | 3.02 | 2.93 | 3.55 | 4.46 | 4.48 | 4.05 | 3.92 | <.0001 |
| Male | 2.26 | 2.35 | 2.35 | 2.42 | 2.47 | 2.70 | 2.82 | 3.12 | <.0001 |
| *Interaction term between year and sex was significant, P < 0.0001. |  |  |  |  |  |  |  |  |  |
